# Bibliometric analysis of research trends on the combination of radiotherapy and PARP inhibitors in solid tumors

**DOI:** 10.3389/fphar.2025.1603573

**Published:** 2025-05-12

**Authors:** Yuxin Chen, Zhengkun Zhang, Rutie Yin, Qingli Li, Wenhao Zhang

**Affiliations:** ^1^ West China School of Medicine, Sichuan University, Chengdu, Sichuan, China; ^2^ State Key Laboratory of Biotherapy and Cancer Center, Sichuan University, Chengdu, Sichuan, China; ^3^ College of Life Sciences, Sichuan University, Chengdu, Sichuan, China; ^4^ Department of Obstetrics and Gynecology, West China Second University Hospital, Sichuan University, Chengdu, China; ^5^ Key Laboratory of Birth Defects and Related Diseases of Women and Children (Sichuan University), Ministry of Education, Chengdu, China

**Keywords:** PARP inhibitors, radiotherapy, radiosensitization, solid tumors, bibliometric analysis

## Abstract

**Introduction:**

Radiotherapy has served as a cornerstone in cancer treatment for over a century. However, the efficacy of radiotherapy is often compromised by the intrinsic and acquired radioresistance of tumors, which can lead to treatment failure and disease recurrence. Recent advancements in preclinical and clinical research have highlighted the potential synergistic efficacy of combining radiotherapy with poly-ADP-ribose polymerase inhibitors (PARPi), offering promising therapeutic avenues for solid tumors. This study employs bibliometric analysis to systematically evaluate the evolution, trends, and intellectual landscape of research on the combination of radiotherapy and PARPi in solid tumors.

**Methods:**

Publications addressing the combination of radiotherapy and PARPi for solid tumors between 2005 and 2024 were retrieved from the Web of Science Core Collection (WOSCC) database. Bibliometric assessments were conducted using VOSviewer and CiteSpace to analyze publication trends, collaborative networks, and research foci.

**Results:**

A total of 901 articles were included. The United States dominated research output, with the University of Texas MD Anderson Cancer Center identified as the most productive institution. Hannah Farmer emerged as the most frequently cited author. Keywords co-occurrence analysis revealed a thematic shift from foundational studies on molecular mechanisms, such as DNA damage response and mechanism of action of PARPi, toward clinical investigations evaluating combination therapy efficacy and safety in trials.

**Conclusion:**

This bibliometric analysis underscores the rapid growth of research on radiotherapy and PARPi combination therapy, with the United States maintaining a leading role due to its extensive scientific infrastructure and collaborative networks. The field has transitioned from mechanistic explorations to translational and clinical applications, reflecting progress toward therapeutic optimization. These findings provide a comprehensive overview of the knowledge structure within this domain and serve as a strategic reference for guiding future research priorities and clinical implementations.

## 1 Introduction

Cancer represents a significant public health challenge worldwide, with approximately 20 million new cases and 9.7 million cancer-related deaths reported annually in the year of 2022 (Bray et al., 2024). Radiotherapy is a cornerstone of cancer treatment, utilized in around 50% of all cancer patients with curative intent (Delaney et al., 2005). The principle of radiotherapy involves the administration of ionizing radiation (IR), either through photon or proton beams, to induce DNA damage and eradicate tumor cells while sparing surrounding normal tissues (Baskar et al., 2008). However, the efficacy of radiotherapy is often compromised by the intrinsic and acquired radioresistance of tumors, which can lead to treatment failure and disease recurrence (Suwa et al., 2021). Radioresistance emerges through two distinct mechanisms: intrinsic alterations in cancer cells (such as genetic or phenotypic adaptations triggered by radiation) or extrinsic protective effects from the tumor microenvironment that shield cancer cells from treatment damage (Jain et al., 2024). Radioresistance leads to cancer relapse, poor treatment response and unfavorable prognosis. The development of effective radiosensitizers that can overcome resistance and minimize toxicity to normal tissues remains a critical goal in oncology research (Hutchinson et al., 2020). Recent advances have highlighted the potential of targeting DNA repair mechanisms, and exploiting synthetic lethality to enhance the therapeutic index of radiotherapy (O'Connor, 2015). However, further investigation is needed to translate these promising findings into clinical practice and improve outcomes for cancer patients.

Poly (ADP-ribose) polymerases (PARP) comprise a family of 17 proteins that play critical roles in various cellular processes, such as DNA repair, chromatin remodeling, stress response, and apoptosis (Rose et al., 2020; Pines et al., 2012; Krishnakumar and Kraus, 2010; Zeng et al., 2024). PARP1, the most extensively studied and well-characterized member of the PARP family, was first identified for its critical role in detecting and repairing single-strand DNA breaks (SSBs) (Fisher et al., 2007). Emerging evidence indicates that PARP1 may also play a role in additional DNA repair pathways, such as nucleotide excision repair (NER) and DNA double-strand breaks (DSBs) repair (Sugimura et al., 2008; Bryant et al., 2005). PARP inhibitors (PARPi) exploit synthetic lethality by targeting cancer cells with homologous recombination (HR) repair defects, especially those with *BRCA1/2* mutations (Bryant et al., 2005). PARP inhibition has been used as monotherapy in the treatment of HR deficient breast cancer, ovarian cancer and prostate cancer with significant improved outcomes (Yap et al., 2019). Besides the study of PARPi monotherapy in HR deficient tumors, PARPi has been investigated extensively as radiosensitizers. Radiation causes DNA damage, primarily SSBs repaired via base excision repair (BER) and more lethal DSBs repaired by non-homologous end-joining (NHEJ) or HR (Rivero Belenchón et al., 2023). PARP inhibition causes SSBs to go unrepaired and collapse into DSBs during replication. Cells then rely on error-prone repair pathways or fail to repair DSBs entirely, amplifying radiation-induced DNA damage and cell death (Li et al., 2023). This synergy makes PARPi effective radiosensitizers. Preclinical studies have demonstrated that PARPi combined with radiotherapy increases cytotoxicity and tumor cell apoptosis. For instance, in ovarian cancer models, the combination of Olaparib and radiotherapy significantly increased apoptosis rates compared to either treatment alone (Bi et al., 2018). Similarly, in breast cancer models, PARPi enhanced the efficacy of proton radiotherapy by increasing DSBs and delaying tumor growth (Ben Kacem et al., 2024). Clinical trials have begun to explore the safety and efficacy of PARPi as radiosensitizers. A phase I study evaluated the combination of Olaparib with carboplatin and radiotherapy in patients with advanced solid tumors, demonstrating manageable toxicity and preliminary efficacy (Loap et al., 2022a). However, results have been inconsistent, with some trials showing improved progression-free survival while others failed to meet their primary endpoints (Kozono et al., 2021; Jiang and Wang, 2022) or showed no significant radiosensitizing effects (Chabot et al., 2017). These discrepancies highlight the need for further research to identify predictive biomarkers and optimize treatment schedules for PARPi-radiotherapy combination.

A comprehensive synthesis of existing studies on the combined use of PARPi and radiotherapy in solid tumors is essential to map the current landscape of knowledge in this field. While previous reviews have outlined the rationale and mechanisms of this combined strategy (Rivero Belenchón et al., 2023; Li et al., 2023; Dungey et al., 2008; Huang and Zhou, 2020; Barcellini et al., 2021; Derby et al., 2022), they remain limited in scope, often omitting broader perspectives and lacking sufficient depth in literature coverage. To address this gap, bibliometric analysis, a method increasingly applied in medical research, offers a powerful tool for macro-level evaluation of large-scale scientific literature (Maggio et al., 2021). By leveraging quantitative metrics and trajectory-tracing capabilities, bibliometrics enables multidimensional exploration of publication trends, collaborative networks, and thematic evolution, thereby uncovering insights that traditional narrative reviews may overlook. So far, no bibliometric analysis has been found concerning combined strategy of PARPi and radiotherapy in solid tumors. Therefore, we conducted a summarized bibliometric analysis on the relevant papers from 2005 to 2024, aiming to explore the framework and directions of this field. The knowledge patterns identified in our study will serve as a foundational framework to guide future research directions in this field.

## 2 Materials and methods

### 2.1 Data acquisition

**FIGURE 1:**
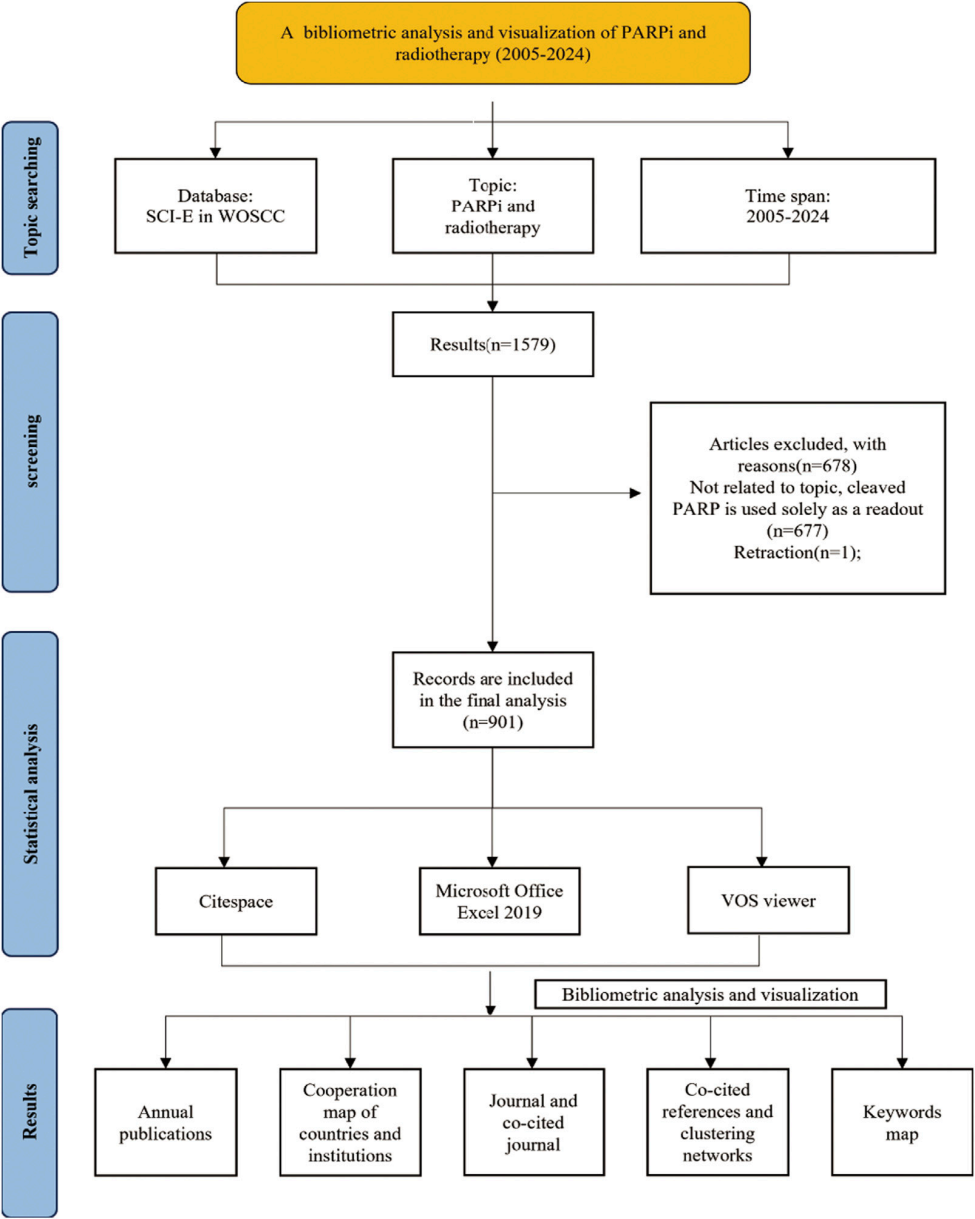
Flowchart of literature collection and selection.

Literature was extracted from the Web of Science Core Collection (WOSCC) database. Data acquisition was conducted on a single day, 31 January 2025, to avoid bias caused by the frequent updates of the WOSCC database. The search terms were as follows: TS = (*cancer* OR *neoplas* OR *tumo* OR *carcinoma* OR *adenocarcinoma* OR *metasta* OR *malignan* OR *sarcoma* OR *melanoma* OR *oncolog*) AND TS = (*parib OR poly (ADP-ribose) polymerase inhibitor* OR PARP inhibitor* OR PARP*) AND TS = (radiotherapy OR radiation therapy OR radiation treatment OR irradiation therapy OR brachytherapy OR chemoradiotherapy OR chemoradiation) NOT TS = (*hodgkin* OR *nonhodgkin* OR *leukemia* OR *leukaemia* OR *lymphoma* OR *myeloma* OR *hematologic* OR *haematologic* OR *hematopoietic* OR *haematopoietic*) NOT TS = (cleaved poly (ADP-ribose) polymerase OR cleaved PARP). The period of studies was set to 2005–2024 encompassing 20 years. The language of the literature was confined to English, and the types of literature were set to articles and reviews (Figure 1).

### 2.2 Bibliometric analysis

In this study, Microsoft Excel 2021, GraphPad Prism (version 9.3.1), VOSviewer (version 1.6.18) and Citespace (6.4 R1) were applied for bibliometric analysis. Tables were generated using Microsoft Excel 2021. VOSviewer is a science mapping tool developed by the Centre for Science and Technology Studies at Leiden University. It enables researchers to create and analyze bibliometric networks, facilitating the visualization of scholarly research patterns and collaborations (van Eck and Waltman, 2010). CiteSpace is a Java-based tool designed for visualizing and analyzing trends and patterns in scientific literature. Developed by Professor Chaomei Chen, it supports progressive knowledge domain visualization and helps identify pivotal moments in a field’s evolution, particularly intellectual turning points and key transitions (Synnestvedt et al., 2005).

## 3 Results

### 3.1 Analysis of general trend

**FIGURE 2:**
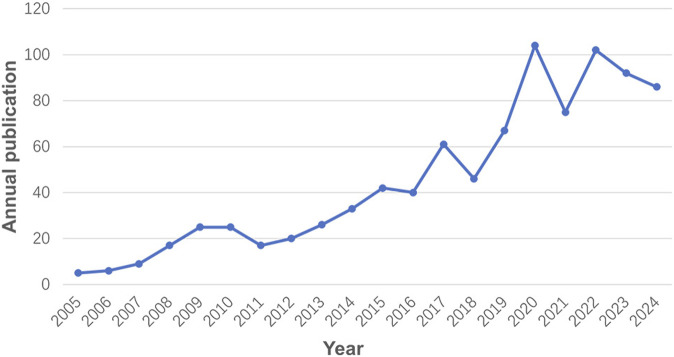
Global trend of publications from 2005 to 2024.

In total, 901 records were finally identified from the database. The annual output has consistently increased with a stepwise growth pattern (Figure 2). Starting with a modest 8 publications in 2005, the annual output surged to 89 by 2024. From 2005 to 2016, the annual output experienced a slow and steady rise under 50. However, after 2016, there was a period of rapid expansion, peaking in 2020 with 105 publications. Despite a minor downturn, the annual output has since stabilized around 90.

### 3.2 Analysis of countries

**FIGURE 3:**
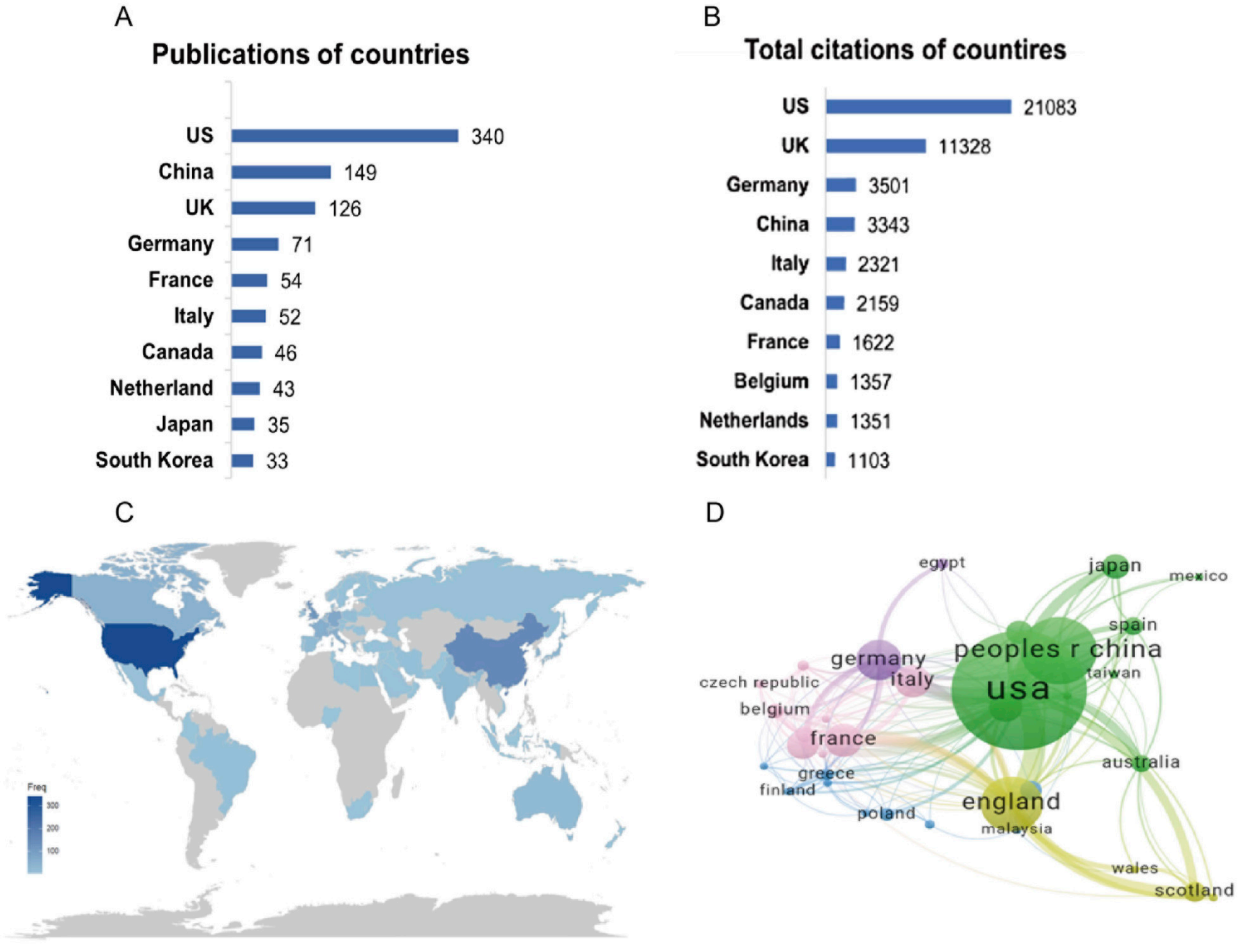
Contribution of countries. **(A)** The top 10 countries in publications. **(B)** The top 10 countries regarding citations. **(C)** A world map of country publications. **(D)** Co-authorship network for countries. Each node corresponds to a country, with node size proportional to publication volume. Connecting lines indicate collaborative relationships between countries, where line thickness reflects the strength or intensity of cooperation.

From 2005 to 2024, a total of 274 countries produced related publications. Figure 3A shows the top 10 countries by publication count, with the United States having the highest number (n = 340), followed by China (n = 149) and the United Kingdom (n = 126). Similarly, in the world map shown in Figure 3C, the United States appears in the darkest shade, indicating the highest frequency of publications. Figure 3B shows that the United States, the United Kingdom, and Germany had the highest total citations (United States: 21,083, United Kingdom: 11,328, Germany: 3,501). The network graph in Figure 3D displays global research collaboration patterns among countries. The most frequent international partnerships occur between the United States and the United Kingdom, followed by the United States and China.

### 3.3 Analysis of institutions

**FIGURE 4:**
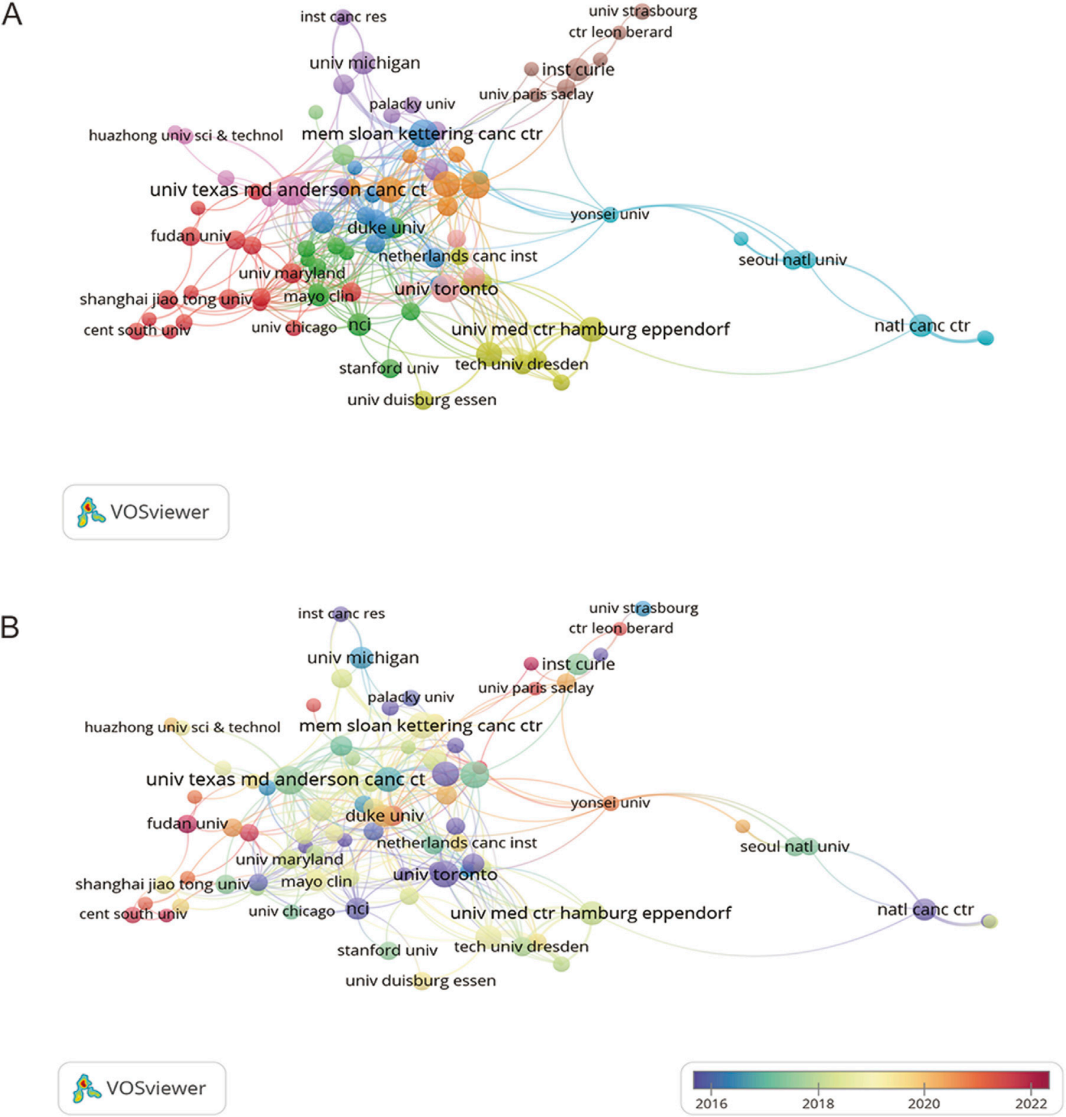
Co-authorship networks for institutions. **(A)** Co-authorship network for institutions. **(B)** Chronological networks for institution co-authorship. Each node corresponds to an institution, with node size proportional to publication volume. Connecting lines indicate collaborative relationships between institutions, where line thickness reflects the strength or intensity of cooperation.

As displayed in Table 1, the University of Texas MD Anderson Cancer Center ranked first regarding the number of documents (n = 27), followed by Memorial Sloan Kettering Cancer Center (n = 24), and University of Oxford (n = 23). Figure 4A visualizes cooperation frequency, indicating the level of collaborative engagement among institutions. The University of Texas MD Anderson Cancer Center, Memorial Sloan Kettering Cancer Center, and University of Oxford are shown to have the most frequent inter-institutional cooperation. Figure 4B depicts the chronological evolution of institutional research output, with a minimum threshold of five publications. The University of Toronto and the University of Sussex, shown in dark purple, demonstrate early engagement in this field. Fudan University, Central South University and University Paris-Saclay were labeled in dark red, suggesting the high activity in recent years.

**TABLE 1:** Top 10 institutions in publications.

Rank	Institution	Country	Publications	Total citations
1	University of Texas MD Anderson Cancer Center	United States	27	1,636
2	Memorial Sloan Kettering Cancer Center	United States	24	3,031
3	University of Oxford	United Kingdom	23	1970
4	University of Toronto	Canada	23	922
5	University of Glasgow	United Kingdom	20	1854
6	Newcastle University	United Kingdom	19	2040
7	University Medical Center Hamburg-Eppendorf	Germany	18	686
8	German Cancer Research Center	Germany	17	522
9	National Cancer Center	Japan	15	459
10	University Michigan	United States	15	2,450

### 3.4 Analysis of journals

**FIGURE 5:**
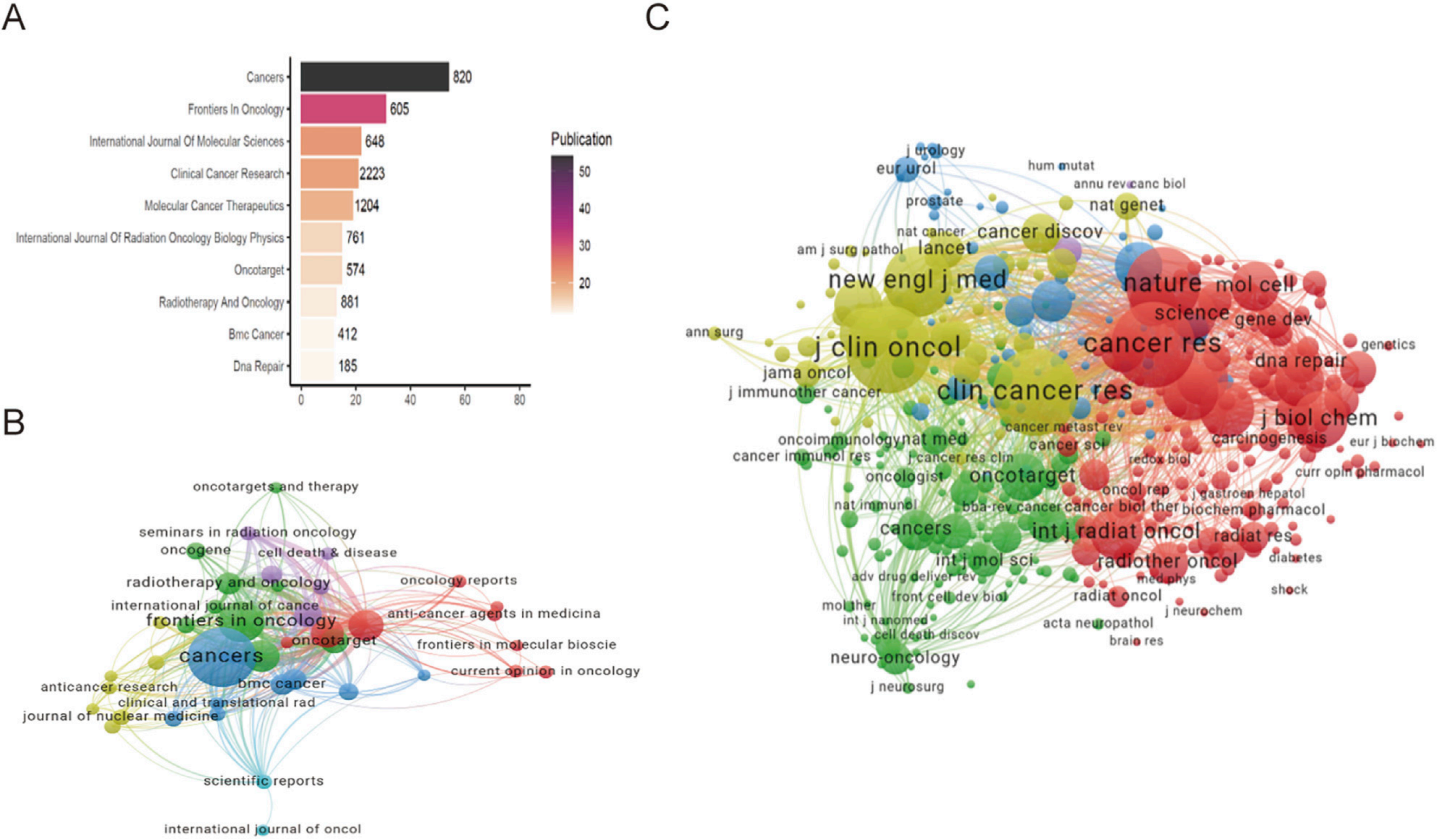
Co-citation and co-cited analysis for journals. **(A)** Top 10 journals in citations and publications. **(B)** Co-citation network. Each node represents a journal in the co-citation network, with node size proportional to the journal’s co-citation frequency. The thickness of connecting lines reflects the strength of co-citation relationships between journals. **(C)** Co-cited journals network. Each node corresponds to a co-cited journal, node size scales with cited frequency. Connecting line thickness indicates the strength of co-citation between journals.

Using VOS viewer, we conducted a co-citation and co-cited journal analysis to identify the most active and influential journals related to the combined therapy of PARPi and radiotherapy in solid tumors. The top three journals by publication count are *Cancers* with 820 citations, *Frontiers in Oncology* with 605 citations and *International Journal of Molecular Sciences* with 648 citations (Figure 5A). The bar color intensity corresponds to publication count, with darker hues indicating higher volumes. The network graph illustrates the relationships between these journals (Figure 5B). *Cancers* has the highest number of publications, and it is connected to many other journals within the local network, showing its central role in the network. Other notable journals include *Frontiers in Oncology*, *Oncotarget and Clinical Cancer Research*, which also have significant connections within the network. Of the top 10 journals, three are from the US, three from Switzerland, three from the Netherlands, and one from the United Kingdom. In addition, *Clinical Cancer Research* has the highest impact factor (IF: 10.4, H-index: 324), while *Oncotarget* has the lowest impact factor (IF: 3.3, H-index: 127). From Figure 5C, it can be seen that the cited journal network consists of four clusters, corresponding to the four colors. *Journal of Clinical Oncology* (2,823 citations), *Clinical Cancer Research* (2,811 citations), *Cancer Research* (2,718 citations), *Nature* (2,143 citations), and *New England Journal of Medicine* (1,830 citations) are the top five most cited journals. These journals represent the most influential and prestigious publications in the field. In the four clusters, the journals in the red cluster primarily focus on basic research fields such as tumor biology and biochemistry. These journal references were selected to critically evaluate PARPi mechanisms and radiosensitization principles, forming the conceptual basis for their own study. The journals in the yellow cluster are primarily clinical oncology journals. They are cited to review classic clinical trials and to provide clinical support for their research.

### 3.5 Analysis of references

**FIGURE 6:**
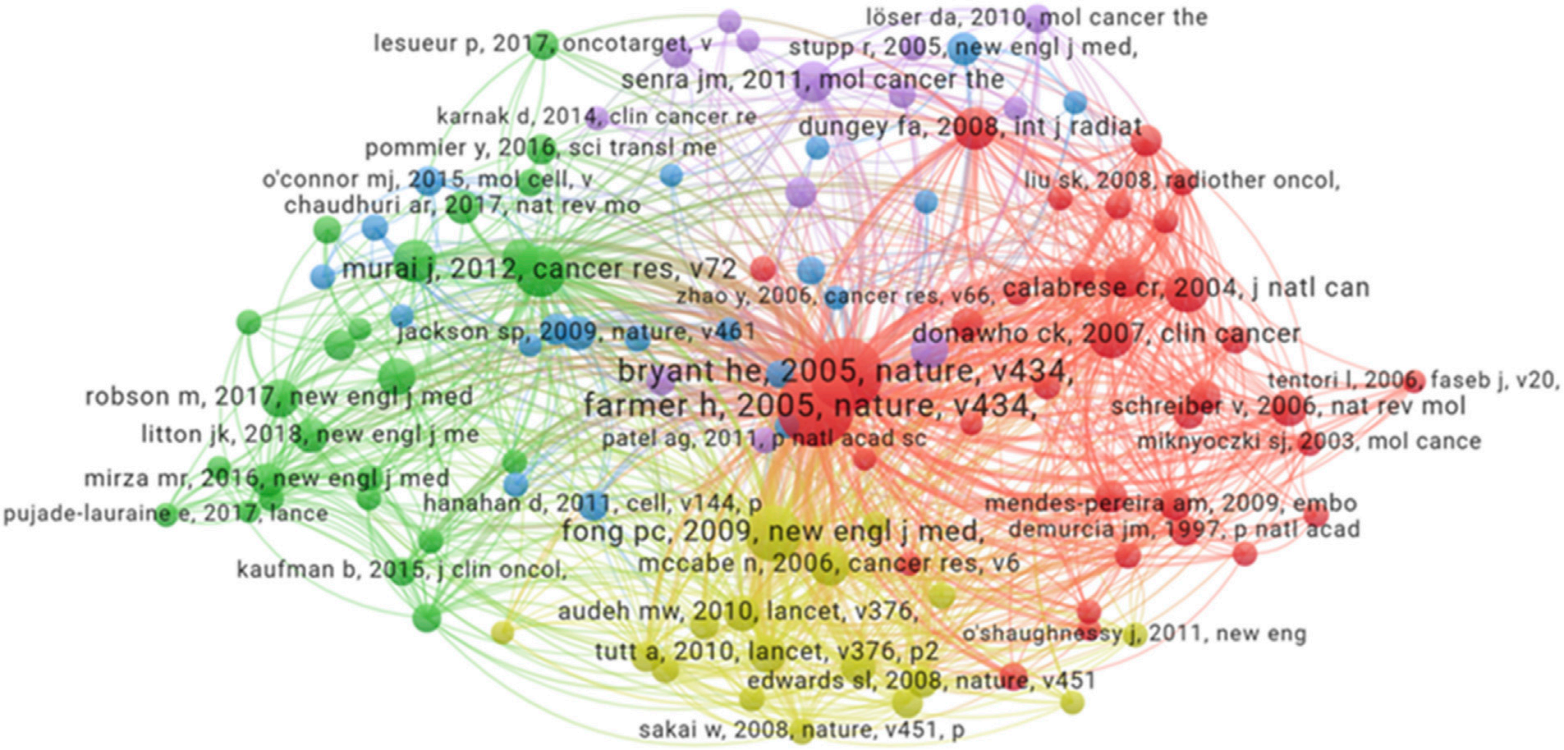
Co-reference network analysis. Each node corresponds to a reference, node size scales with reference frequency. Connecting line thickness indicates the strength of co-occurrence relationships between references.

A total number of 42,839 related references in the field of were included in the co-reference analysis. Each node represents a reference, and the node size corresponds to the number of citations (Figure 6). Node colors represent distinct research clusters or subfields within the domain. The graph highlights key influential papers, such as the one published in *Nature* titled “*Targeting the DNA repair defect in BRCA mutant cells as a therapeutic strategy*” by Farmer et al. (2005), which has the highest citation of 276, and the study in *Nature* titled “*Specific killing of BRCA2-deficient tumours with inhibitors of poly(ADP-ribose) polymerase*” by Bryant et al. (2005) which has the citation of 248. These two papers are foundational works in the field, as they were the first to elucidate the role of *BRCA2* in regulating DNA repair and to demonstrate how the impairment of this function in cancer cells can be leveraged for therapeutic purposes. This discovery laid the groundwork for the development of PARPi. This visualization helps in identifying seminal works and their impact on the broader research community.

### 3.6 Analysis of keywords

**FIGURE 7:**
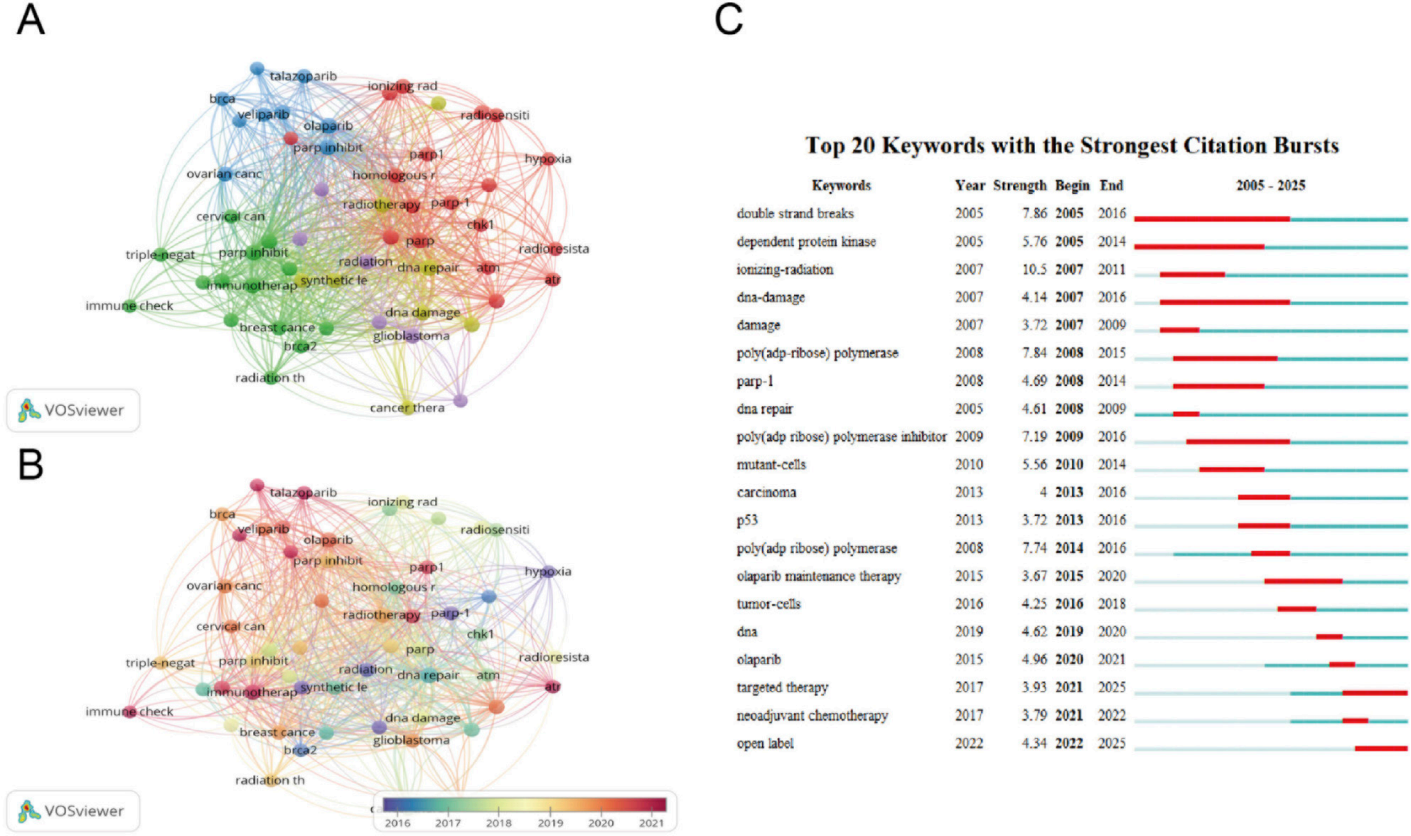
Co-occurrence networks for keywords. **(A)** Clusters for keywords co-occurrence. **(B)** Chronological clusters for keywords co-occurrence. Each node corresponds to a distinct keyword, node size scales with keyword frequency. Connecting line thickness indicates the strength of co-occurrence relationships between keywords. **(C)** Top 20 keywords with the strongest citation bursts. Year indicates the publication year of the keyword. Strength reflects the emergence intensity, with higher values denoting greater scholarly attention. Begin and end mark the temporal boundaries of the keyword citation peak period, corresponding to the red highlighted segments in the visualization.

The cluster visualization of 1,651 keywords extracted from the documents was displayed in Figure 7A. The minimal keyword occurrence was set to 10 to ensure readability. The red cluster consisted of PARP, PARP1, apoptosis, HR, ATM, ATR. This cluster represented the potential mechanisms of PARP1 involved in the DNA damage response. The major terms of the blue cluster included Olaparib, PARPi, Veliparib, Niraparib, ovarian cancer, Talazoparib, in which the main focus of this cluster was PARPi and maintenance therapy. The green cluster was composed of PARPi, chemotherapy, immune checkpoint inhibitor, cisplatin, breast cancer and cervical cancer. The main focus of this cluster was clinical utility of PARPi in combination therapy. The purple cluster primarily focused on radiation, glioblastoma, indicating research related to glioma. In the yellow cluster, DNA repair, synthetic lethality, radiosensitizer were the main nodes included, and this cluster indicated the potential of radiosensitization by PARPi. Figure 7B shows the temporal evolution of keywords according to the gradient of color, with darker purple indicating earlier studies and dark red representing more recent active topics. The purple to green cluster includes terms such as PARP1, radiation, hypoxia, synthetic lethality, focusing on mechanism of PARPi causing cell death, which were published at the average year of 2016–2017. The red cluster includes immunotherapy, Veliparib, Talazoparib, and radiotherapy, indicating the potential application of PARPi combined with radiotherapy in the clinical management, which were published in the year of 2020–2021 at average. Keyword burst detection in Citespace identifies terms that experience a sudden surge in frequency over a specific time period. This method allows researchers to track trends in PARPi combined with radiotherapy studies, uncovers shifts in research focus, and pinpoints emerging topics in the field. The keyword burst analysis reveals an evolution in research focus within this field, transitioning from foundational studies to clinical applications, as illustrated in Figure 7C. For example, the first burst keyword is DSBs with a strength of 7.86 and time span 2005–2016, indicating that the research focus was on the molecular mechanisms of DNA damage. In contrast, the most recent burst keywords are targeted therapy (strength 3.93, time span, 2021–2025) and open label (strength 4.34, time span, 2022–2025), suggesting that the research focus has shifted to clinical trials.

## 4 Discussion

To our knowledge, this study represents the first bibliometric analysis examining the intellectual foundation and research frontiers of PARPi combined with radiotherapy. Using bibliometric and visual analysis methods, we evaluated global research trends in this field, including publication outputs, contributing countries, institutions, journals, and keywords distributions. Our search strategy identified 901 relevant publications indexed in the Web of Science from 2005 to 2024. These articles were published across 342 academic journals and authored by 5,434 researchers spanning 63 countries and regions.

### 4.1 General information

Annual publication trends provide a clear indicator of progress in a research field. Over the past 2 decades, studies on PARPi combined with radiotherapy for solid tumors have exhibited a biphasic growth pattern. Early phase (2005–2016): annual publications remained below 50, but the field grew rapidly at an average rate of 43.47% per year. Recent phase (2017–2024): output surged to an average of 79 publications annually, though growth moderated to 15.58% per year, reflecting maturation of the field. This trend highlights continued exploration of radiotherapy combined with PARPi. The marked increase in both publications and citations underscores rising scientific interest. We anticipate further advancements in radiotherapy-PARPi combination for solid tumors in the near future.

Our analysis reveals the United States’ dominant role in this research field, demonstrating superior productivity and influence across three key metrics: publication volume (accounting for 38.01% of global output), citation impact, and international collaboration. This leadership is further evidenced by institutional rankings, with United States institutions comprising three of the top ten, including the top two positions, and producing the most prolific authors in the field. Notably, the University of Texas MD Anderson Cancer Center emerges as a particularly significant contributor. These findings not only confirm the United States’ current preeminence in PARPi-radiotherapy research but also suggest this domain will maintain its position as a high-priority area for continued scientific investigation and development.

Journal influence is typically measured by Impact Factor (IF) and Journal Citation Reports (JCR), which divide journals into four quartiles based on IF (Saginur et al., 2020). Among the top 10 journals on radiotherapy-PARPi combination, *Cancers* (54 publications) is the most productive, followed by *Frontiers in Oncology* (31 publications) and *International Journal of Molecular Sciences* (22 publications). Except *Oncotarget*, all top 10 journals are Q1 or Q2, with *Clinical Cancer Research* having the highest IF of 10.4. Notably, despite publishing only 21 articles on this topic, *Clinical Cancer Research* has garnered 2,223 citations, achieving the highest average citation rate, which is a testament to the journal’s significant academic influence in this field. These journals demonstrate substantial scholarly influence and rigorous quality standards, positioning them as essential resources for researchers identifying core publications in this discipline.

In bibliometric analysis, co-occurrence network mapping reveals research hotspots through keywords associations (Figure 7A), while temporal clustering visualization (Figures 7B, C) tracks the evolution of emerging research fronts (Xiao et al., 2017). The high-frequency keywords of PARPi and radiotherapy combination research (Figures 7A,B) included DNA repair, radiotherapy, PARPi, PARP, Olaparib, immunotherapy, etc., which were regarded as the hotspots in this filed. As time goes on, emerging topics gradually change to targeted therapy, open label, immunotherapy, etc., suggesting that the research focus has shifted to clinical investigation and immunotherapy. Keywords clustering analysis not only maps the discipline’s intellectual architecture but also identifies its evolving research frontiers (Qin et al., 2020). Cluster analysis showed five main clusters in the PAPRi-radiotherapy combination field, including the mechanism of DNA damage repair, the mechanism of action of PAPRi, the research of glioblastoma, various commercially available PARPi, investigation in cervical cancer, breast cancer, and immunotherapy, which represent the main hotspots of PARPi-radiotherapy combination research to some extent.

### 4.2 Mechanism of function

The radiobiological cascade after IR initiates with SSBs that undergo error-prone repair, progressing to lethal DSBs during DNA replication (Bondar and Karpichev, 2024). This cumulative genomic instability overwhelms the DNA damage response capacity of malignant cells, triggering mitotic catastrophe and apoptotic cell death. Mechanistically, the radiosensitizing effect of PARPi operates via a replication-dependent synthetic lethality mechanism: persistent PARP inhibition converts radiotherapy-induced SSBs into unrepaired DSBs during S-phase progression, causing replication fork collapse and chromosomal disintegration (Dungey et al., 2008; Noël et al., 2006). This therapeutic synergy is especially pronounced in HR-deficient malignancies, where defective *BRCA*-mediated DSBs repair intensifies genomic instability, further enhancing the therapeutic index (Lesueur et al., 2017).

The mechanistic basis of PARPi-mediated radiosensitization was initially characterized in pivotal studies by Chalmers et al. (2004). Their experiment tested the effect of PARPi on low-dose radiation in several cell lines and the results showed the radiosensitizing effect of PARPi in highly actively dividing tumor cells. Beyond direct cytotoxicity, radiotherapy demonstrates multimodal immunomodulatory effects through two key mechanisms: induction of immunogenic cell death and tumor microenvironment reprogramming, which collectively enhance innate antitumor immunity (McLaughlin et al., 2020). These effects complement established PARPi mechanisms, particularly cGAS-STING pathway activation and subsequent cytotoxic CD8^+^ T-cell recruitment (Pilger et al., 2021). These preclinical experiments establish a solid foundation for subsequent clinical trials. Numerous Phase I clinical trials have investigated the combination of PARPi and radiotherapy across a range of solid tumors, including prostate cancer (Quinn et al., 2023; Pan et al., 2023), triple-negative breast cancer (Loap et al., 2022b), glioblastoma (Derby et al., 2024; Stefan et al., 2025), non-small cell lung cancer (de Haan et al., 2021), brain metastases from lung and breast cancer (Mehta et al., 2015), diffuse pontine glioma (Baxter et al., 2020), pancreatic cancer (Tuli et al., 2019) and soft-tissue sarcoma (Sargos et al., 2025) demonstrating that these combinations are generally well tolerated.

### 4.3 Outlook

Clinical trials have demonstrated the safety of PARPi combined with radiotherapy, with early evidence of clinical benefit, warranting further evaluation in larger trials. Phase II clinical trials investigating PARPi combined with radiotherapy in advanced sarcoma (NCT06074692), oligometastatic ovarian cancer (NCT05990192), breast cancer (NCT04837209, NCT03598257), and head and neck squamous cell carcinoma (NCT05784012) are ongoing, with positive outcomes expected. To advance this field, three strategic directions warrant prioritized investigation. First, therapeutic optimization demands rigorous clinical validation to maximize tumor suppression while minimizing systemic toxicity. Although no randomized trials have directly compared PARPi head-to-head, and only indirect cross-comparisons can be drawn from the available literature, the Food and Drug Administration (FDA) approved PARPi (Niraparib, Olaparib, Rucaparib, and Talazoparib) and Chinese National Medical Products Administration (NMPA) approved PARPi (Pamiparib, Fluzoparib) exhibit several overlapping adverse effects (Zeng et al., 2024; Friedlander et al., 2023; LaFargue et al., 2019). Dai et al. performed a network meta-analysis to systematically evaluate the safety profiles and hematological toxicities of PARPi, utilizing data from 27 randomized controlled trials and a pharmacovigilance database (Dai et al., 2024). The results revealed that Olaparib had a significantly lower risk of any grade 3–5 adverse effects than Niraparib, Rucaparib, Fluzoparib and Talazoparib. Moreover, based on the surface under the cumulative ranking curve (SUCRA) rankings, Olaparib was the safest PARPi in terms of risk of hematological adverse effects (Dai et al., 2024), supporting its high potential for combination with radiotherapy. Beyond commercially available PARPi, emerging selective PARPi candidates from preclinical development demonstrate promising potential to widen the therapeutic window through enhanced tumor specificity and reduced off-target effects. Second, precision oncology implementation demands biomarker-driven patient stratification. Identification of molecular signatures predictive of combination therapy response remains paramount for optimizing clinical benefit. BRCA status serve as key predictors of PARPi responsiveness, consequently enhancing radiosensitizing effects. However, besides ovarian, breast, and prostate cancers, *BRCA1/2* mutations are rare in other tumor types (Szentmartoni et al., 2024). This highlights the importance of identifying more prevalent genetic alterations that could enable PARPi-mediated radiosensitization in BRCA-wild-type solid tumors. Genetic and pharmacological alterations of specific proteins that can induce a state of “BRCAness” or HR-deficiency in BRCA-proficient cancers have the potential to broaden the application of PARPi (Singh et al., 2020). Finally, treatment parameter standardization must be systematically investigated, including definitive optimization of dosing regimens, administration schedules, and temporal coordination between radiation fractions and PARPi cycles.

### 4.4 Limitation

This study provides a comprehensive synthesis of 20 years of research on radiotherapy and PARPi combination therapies for solid tumors. However, several methodological limitations should be noted. Language bias: restricting the analysis to English-language publications may overlook significant contributions from non-English sources, potentially skewing geographic and institutional representation. Database limitations: sole reliance on the Web of Science Core Collection (WOSCC) risks excluding relevant studies indexed in other major databases. Temporal constraints: while the two-decade span captures key trends, the 2005 cutoff excludes earlier foundational work and very recent breakthroughs. Methodological bias: bibliometric analysis inherently prioritizes quantitative metrics; future research could integrate qualitative approaches to deepen thematic insights. The analytical outputs of bibliometric tools such as VOSviewer and CiteSpace are influenced by user-selected parameters, including citation thresholds, clustering resolutions, and temporal segmentation, etc. These parameter choices may introduce systematic biases into the analysis. A notable example is the set of minimum citation thresholds for node inclusion, which risks omitting recently published yet influential works, consequently favoring more established publications in the resulting network visualization. These limitations highlight opportunities for more inclusive and multidimensional analyses in future meta-research.

## 5 Conclusion

Global scientific engagement in PARPi-radiotherapy combination for solid tumors has evolved markedly over 2 decades. The growth in annual publications underscores this field’s expanding scientific and clinical relevance. The United States emerged as the predominant contributor, leading in research output, institutional participation, and author productivity. Analysis of keywords dynamics revealed a paradigm shift from fundamental DNA damage response mechanisms to clinical translation strategies in recent years. This bibliometric investigation systematically maps the field’s intellectual architecture while delivering a systematic framework for understanding current and emerging research priorities.
